# What Do the General Public Know about Infertility and Its Treatment?

**DOI:** 10.3390/ejihpe14080141

**Published:** 2024-07-24

**Authors:** Lewis Nancarrow, Anuthi Fernando, Lucy Hampton, Courtney Murray, Dharani K. Hapangama, Nicola Tempest

**Affiliations:** 1 Department of Women’s and Children’s Health, Centre for Women’s Health Research, Institute of Life Course and Medical Sciences, University of Liverpool, Member of Liverpool Health Partners, Liverpool L8 7SS, UK; lewis.nancarrow@nhs.net (L.N.); hlbferna@liverpool.ac.uk (A.F.); hllhamp4@liverpool.ac.uk (L.H.); hlcmurra@liverpool.ac.uk (C.M.); dharani@liverpool.ac.uk (D.K.H.); 2 Hewitt Centre for Reproductive Medicine, Liverpool Women’s NHS Foundation Trust, Liverpool L8 7SS, UK; 3 Liverpool Women’s NHS Foundation Trust, Member of Liverpool Health Partners, Liverpool L8 7SS, UK

**Keywords:** fertility, knowledge, education, assisted reproductive technologies, ART, in vitro fertilisation, IVF

## Abstract

Rates of infertility are rising, and informed decision making is an essential part of reproductive life planning with the knowledge that ART success decreases dramatically while a woman’s age increases and that high costs can often be incurred during fertility treatment. We aimed to determine the current knowledge of infertility and its treatments in the general public through an online survey. We received 360 complete responses. The average age of respondents was 35 years with most respondents being female (90%), heterosexual (88%), white (85%) and university educated (79%). Of the total, 49% had children and 23% had a condition that affects their fertility; 41% had concerns about future fertility and 78% knew someone who had had fertility treatment. Participants’ understanding of basic reproductive biology and causes of infertility varied with correct responses to questions ranging from 44% to 93%. Understanding of IVF outcomes was poorer with only 32% to 55% of responses being correct, and 76% of respondents felt that their education in fertility was inadequate. This survey highlights the inconsistencies in the general public’s understanding of infertility in this relatively educated population. With increasing demands on fertility services and limited public funds, better education is essential to ensure patients are fully informed with regard to their reproductive life planning.

## 1. Introduction

Infertility is defined as a disease of the reproductive system with a failure to achieve a clinical pregnancy after 12 months or more of regular unprotected intercourse [1]. Infertility is common, with one in six heterosexual couples struggling to conceive [2], with a global prevalence of over 48 million couples affected [3]. In developed countries fertility rates continue to decrease, partly due to better access to contraception, but also due to increasing maternal age, increasing levels of obesity and continued negative stigma towards young parenthood [4,5,6,7]. The average maternal age in England and Wales has increased from 26.4 years in 1974 to 30.9 in 2021 [8], with this trend being replicated in other developed countries [9,10,11]. Infertility can lead to distress, depression, discrimination and ostracism with associated costs to individuals and society being huge [3]. 

Despite its prevalence, the perception of and knowledge about infertility amongst the general public continues to be poor. One of the first surveys on infertility perceptions in 2000, which included 8194 adults from eight different countries found that 62% of respondents did not perceive infertility to be a disease and their awareness of the definition and incidence of infertility was low [12]. Other subsequent surveys have failed to show improvements in knowledge despite the increase in demand for assisted reproductive treatments (ARTs) [9,10,13,14,15]. 

The primary aim of our study was to assess the general knowledge about infertility in the UK. The secondary aim was to evaluate whether a difference in age, gender, education or sexual orientation accounted for any significant differences in an individual’s knowledge of infertility. 

## 2. Materials and Methods

### 2.1. Ethics 

This online anonymous questionnaire was approved by the University of Liverpool’s Institute of Life Course and Medical Sciences Research Ethics Committee (ref—11997). 

### 2.2. The Survey 

An initial literature review of previous surveys on infertility knowledge was performed, this informed our final survey, which included 40 questions (Supplementary File S1). The survey was divided into five main subsections: demographics, personal fertility history, knowledge of basic fertility, causes and risk factors and knowledge of in vitro fertilisation (IVF) as a treatment option. The demographic data collected from the questionnaire included age, gender, sexual orientation, ethnicity, country of residence and their highest level of education. The personal fertility history section included questions about previous fertility treatment and if they would consider fertility preservation methods in the future. The section on knowledge of basic fertility biology was included to highlight areas of knowledge that are incomplete or incorrect. Questions related to causes and risk factors for infertility determined the participants’ knowledge with regard to lifestyle factors or conditions that impacted fertility. Finally, knowledge related to IVF allowed us to determine the respondent’s understanding of IVF treatments and their success rates. 

The survey was advertised on social media through the online survey tool SurveyHero (www.surveyhero.com). A participant information sheet (PIS) was included for respondents to read prior to completing the survey and the first question in the survey confirmed the participants consented to complete the questionnaire. Only the participants who provided consent were eligible to progress and complete the questionnaire. Questionnaires were included in the analysis if all questions were answered and the data provided by the respondents fulfilled the inclusion criteria of being over the age of 18 years. Survey responses were collected over a 3-month period. 

### 2.3. Statistical Analysis

This survey was not designed as a comparative study to test a hypothesis, and thus, power calculation was not appropriate. Therefore, in line with our research, we report summary statistics of the data obtained from the survey. Where possible, the Statistical package for the Social Sciences (SPSS) for Windows (Version 26; IBM Corporation, New York, NY, USA) was used to analyse categorical data using the chi squared test or the students paired *t*-test for continuous data. 

## 3. Results

There were 428 responses to the survey; 68 were excluded due to incomplete responses and 1 respondent was excluded due to being 16 years old. The final number of complete responses for analysis was 360. 

Most respondents resided in the United Kingdom (336, 93.3%), were white (305, 84.7%) and had a university education (283, 78.6%) (Table 1). 

### 3.1. Personal Fertility History

Of those surveyed, 190 (52%) did not have a child, 52 (14%) of participants were trying to conceive and 77 (21%) had a known condition that could affect their ability to conceive in the future. Of the participants, 151 (42%) were concerned about having a child in the future and the majority of the participants (278, 77%) knew someone who had gone through fertility treatment previously, with 51 (14%) having had fertility treatment (Table 2). 

### 3.2. Basic Fertility Knowledge

The number of participants that were correctly able to define the duration of time needed to have passed prior to an infertility diagnosis was 211 (58.6%) (12 months). The majority of participants were able to correctly identify how many days were in the average menstrual cycle (335, 93.1%) and the ovulation window (265, 73.6%) and the most likely day of ovulation (233, 64.7%). Optimal frequency of intercourse was answered correctly by 63.5% of participants (229). Respondents’ knowledge about the lifespan of sperm in the female reproductive tract and oocyte lifespan following ovulation varied. Although most participants were aware that a female’s age has an impact on her fertility potential (340, 94.4%), knowledge of when the fertility started to decrease varied significantly between respondents (Table 3). 

Participants were less aware that male fertility was affected by age with only 164 (46%) choosing the correct response. Of those that correctly identified that male fertility decreases with age, only 79 (48%) correctly answered that its deterioration starting between 40 and 45 years old. 

Out of the nine questions in this section the average number of correct responses was 3.9/9 (43%).

### 3.3. Causes of and Risk Factors for Infertility

Participants were given a list of potential causes of infertility in both women and men and were able to pick multiple options. The results can be seen in Table 4 and Table 5.

The main cause of tubal blockage (chlamydia) was identified by 210 (58.3%) participants. The majority (266, 73.9%) also answered correctly that infertility causes are equally spread amongst both male and female partners. Out of this section the average number of correct responses was 26.1/31 (84%). 

### 3.4. Knowledge of IVF 

Understanding of IVF was poor across all participants, with the highest correct response rate being 55% (n-198) (cost of IVF GBP 1500–GBP 5000). Only 114 (32%) of participants were aware that there were 48 million couples affected by infertility worldwide, 139 (38.6%) participants correctly identified the current IVF success rate of 32% and 166 (46.1%) correctly answered that 8 million children have been born through IVF. 

Similarly, 140 (38.9%) participants were aware that the average number of IVF cycles funded by the NHS is two. There was strong agreement that IVF should be funded by the NHS (326, 90.6%) and that two or three cycles of IVF should be funded (227, 63%). In this section, the average number of correct responses was 2.1/5 (42%). 

When asked about whether participants had received substantial teaching on fertility in school/college, most felt their teaching was insufficient (272, 75.5%). 

In total, the mean number of correct responses per participant was 34.7/47 (74%). When removing the causes of fertility, the mean number of correct responses dropped to 8.6 out of 16 questions (54%). 

### 3.5. Subgroup Analyses

There was no difference in responses by different age groups. When grouped into gender, males were less likely to identify the correct average menstrual cycle length (*p* < 0.001); otherwise, there was no difference between male and female responses. As there were only two non-binary participants in the survey, they were not included in the statistical analysis. When comparing education levels, the only question that showed a statistical difference in responses was on which out of a couple were more likely to be the cause of infertility (*p* = 0.011). As there was only one participant who had no formal education, they were not included in the statistical analysis. 

All groups thought that the cause of infertility was both the male and female partner in equal measure; however, when equal was excluded as an answer, those who had a secondary-level education felt females (*n* = 4) were more likely than males (*n* = 0) to be the cause of infertility. To a lesser extent, university-educated participants felt that females (*n* = 48) were more likely than males (*n* = 25) to be the cause of infertility. Those with a vocational education felt that males (*n* = 6) and females (*n* = 6) were both as likely to be the cause of infertility. 

When comparing answers between groups with different sexual orientation there were a number of statistically significant differences in the groups’ responses. Heterosexual participants were less likely to think male depression impacted fertility (*p* = 0.018, Table 6) and homosexual participants were more likely to think males having multiple sexual partners would affect fertility (*p* < 0.001, Table 6). 

## 4. Discussion

This contemporary survey updates the 20-year-old previous worldwide survey [12] on the public perception of fertility. Whilst there are still some areas for improvement regarding particular responses, there seem to be an encouraging improvement in the participants understanding of infertility in comparison to previous surveys [9,10,11,12,13,14,15], with the average participant answering 74% of the questions correct. 

When reviewing this cohort’s basic knowledge of fertility, the correct responses to the questions ranged from 39% (how long is an oocyte capable of being fertilised by a spermatozoa?) to 93% (advancing age of females affects fertility). Superficial knowledge related to the menstrual cycle, including cycle length (93%), ovulation window (74%) and ovulation day (65%) had a high number of correct responses; however, participants responses regarding lifespan of the oocyte (39%) or sperm (44%) revealed poor knowledge. Similarly to other surveys, a high proportion of our participants were aware that female age affects fertility (94%); however, the effect of male age on fertility was not similarly well understood with only 46% answering correctly. Seventy four percent of the participants responded that in those struggling to conceive, both the male and female partner were equally likely to be the cause of infertility (74%). However, this awareness of female factors and apparent lack of awareness of the impact of male age on fertility is likely due to the focus of treatment for infertility still being on females, even in cases of male infertility [16,17]. As a consequence, male infertility is discussed less in the public domain, often leading to a lack of awareness regarding the male role in infertility and conception [16,18]. 

Our participants showed a poor understanding of the definition of infertility with 59% answering correctly; however, in comparison to previous studies, this suggests a slight improvement [9,10,14]. This lack of understanding can impact future patient care. In some cases, couples will delay treatment, potentially reducing their chances of conception with future treatment [19,20], whereas others may seek investigations and treatments too early, incurring additional costs to themselves and to the health service [21]. 

It was reassuring to see that participants were aware of the potential risks factors that impact fertility including smoking, obesity and alcohol. However, despite the high mean score in this subsection, there were a number of incorrect answers. Worryingly, a third of participants thought that hormonal contraceptives, previous termination of pregnancy, recurrent urinary tract infection and candida infections impact fertility prospects despite evidence to the contrary [22,23,24,25,26,27]. 

Despite 77% of participants knowing people who have gone through fertility treatment, the average score knowledge of IVF was 42%. The poor score in the knowledge of IVF was surprising with the high prevalence of fertility treatment. The knowledge was equally poor, amongst participants who claimed to know others who had fertility treatment or had been through treatment themselves (n-279, 77.5%), thus, highlighting the need for further education. Despite the lack of knowledge regarding the IVF process, there was strong support for IVF treatment with over 90% of participants advocating for NHS-funded treatments. This positive outcome has been mirrored in many other previous studies [10,12,28], with most agreeing with the current National Institute for Health and Care Excellence (NICE) recommendation [29] for three funded cycles of IVF treatment. 

Interestingly, in the subgroup analysis, there were very few differences noted between groups. Resoundingly, the majority of participants in this cohort felt that they had insufficient education on basic fertility and treatment. Our findings highlight the need for a further review of the current secondary education exposure to fertility teaching. Relationship and sex education became mandatory in schools in England and Wales in 2020. It would be interesting to understand if responses to our questionnaire would be improved as a result of this new mandatory requirement of the curriculum.

In relation to fertility education specifically, one key strategy may be the increased integration of reproductive life planning (RLP) into both secondary and tertiary education settings in the UK. RLP aims to encourage individuals to reflect on their reproductive plans, and what actions to take to realise them [30]. A combination of overestimations of IVF success rate, a reduced awareness of infertility epidemiology, an increasing age of childbearing and several respondents who would not consider fertility preservation methods is an indication for greater fertility awareness needs. Integration of these aspects in a quality RLP tool to be used in secondary and tertiary education settings may facilitate this education process.

### Limitations

This survey was answered predominately by white, heterosexual, university-educated females living in the UK. Therefore, it is unfortunately not representative of the general wider population and further surveys including respondents from a wider demographic background are required to appreciate a more representative sample of participants. However, since respondents in our survey would have traditionally been expected to be more aware about their own fertility, their responses demonstrating poor overall knowledge further highlights the deficiencies and inconsistencies in the current education related to fertility. Future studies should explore the information sources currently used by the general public regarding fertility to ensure that we prevent the spread of misinformation and inform and empower people appropriately through reliable sources of information that are universally accessible and suitable despite their level of technological literacy. 

## 5. Conclusions

This online survey highlights the significant inconsistencies in the understanding of infertility among responders from the UK. With increasing demands on fertility services and limited public-funds allocated for infertility treatment, patients will benefit from being well informed about how and when to start a family if desired, costs associated with fertility treatments and the options for fertility preservation. The complexities that exist with advanced fertility treatments and the limited success rates with IVF make it essential for couples to be well informed when making decisions regarding their fertility treatments.

## Figures and Tables

**Table 1:** Demographics.

Age in Years, Mean (Range)	34.9 (18–75)
Gender, *n* (%)	
Female	324 (90)
Male	32 (8.9)
Non-Binary	2 (0.6)
Prefer not to say	1 (0.3)
Not reported	1 (0.3)
Sexual orientation, *n* (%)	
Asexual	2 (0.6)
Bisexual	27 (7.5)
Heterosexual	315 (87.5)
Homosexual	6 (1.7)
Other	2 (0.6)
Prefer not to say	7 (1.9)
Not reported	1 (0.3)
Ethnicity, *n* (%)	
Asian	29 (8.1)
Black	9 (2.5)
Mixed	16 (4.4)
Other	1 (0.3)
White	305 (84.7)
Level of education, *n* (%)	
No formal education	1 (0.3)
Secondary education	35 (9.7)
Vocational education	41 (11.4)
University education	283 (78.6)

**Table 2:** Personal fertility history.

	Number (%)
Previous children?	
Yes	177 (49.2)
No	183 (50.8)
Trying to conceive?	
Yes	47 (13.1)
No	292 (81.1)
Not applicable	19 (5.3)
Not reported	2 (0.6)
Have a condition affecting fertility?	
Yes	83 (23.1)
No	196 (54.4)
Not sure	81 (22.5)
Concerned about ability to conceive?	
Yes	147 (40.8)
No	158 (43.9)
Not applicable	55 (15.3)
Know people who have had fertility treatment?	
Yes	279 (77.5)
no	81 (22.5)
Had fertility treatment previously?	
Yes	55 (15.3)
No	305 (84.7)
If you had fertility treatment, what did you have? *	
Ovulation induction	13 (22.0)
Intrauterine insemination	1 (1.7)
IVF/ICSI	43 (76.3)
Would consider fertility preservation methods?	
Yes	105 (29.2)
Maybe	84 (23.3)
No	72 (20.0)
Already had	12 (3.3)
Not applicable	87 (24.2)

* Some people had more than one treatment.

**Table 3:** Basic fertility knowledge. Bold shows correct answer.

What is the Duration in the Definition of Infertility?	
Duration	Number (%)
6 months	30 (8.3)
**12 months**	**211 (58.6)**
15 months	5 (0.1)
18 months	35 (9.7)
24 months	79 (21.9)
How long can sperm survive in the female reproductive tract?	
Days	Number (%)
1	48 (13.3)
3	134 (37.2)
**5**	**160 (44.4)**
10	10 (2.7)
15	8 (2.2)
Following ovulation, how long is an oocyte capable of being fertilised by a spermatozoa?	
Hours	Number (%)
6–12	24 (6.7)
**12–24**	**139 (38.6)**
24–48	112 (31.1)
48–72	70 (19.4)
72–96	15 (4.2)
What is the age when female fertility starts to decline?	
Age (years)	Number (%)
<20	1 (0.2)
20–29	31 (8.6)
30–34	87 (24.2)
**35–39**	**176 (48.9)**
40–44	37 (10.3)
>45	5 (1.4)
No answer	23 (6.4)

**Table 4:** Which of the following factors can negatively impact female fertility?

Age (Years)	Number Correct (%)
**Smoking**	**341 (94.7)**
**Depression/mental health**	**229 (63.6)**
**Being overweight**	**337 (93.6)**
**Polycystic ovarian syndrome**	**345 (95.8)**
**Endometriosis**	**332 (92.2)**
Recurrent yeast infections	223 (61.9)
Previous urinary tract infections	233 (64.7)
Eating red meat	315 (87.5)
Previous termination of pregnancy	246 (68.3)
Multiple sexual partners	318 (88.3)
Previous hormonal contraceptives	246 (68.3)
Frequent masturbation	359 (99.7)
Breast size	358 (99.4)
Height	358 (99.4)
Genital size	346 (96.1)

Bold shows factors that affect female fertility.

**Table 5:** Which of the following factors can negatively impact male fertility?

Age (Years)	Number Correct (%)
**Smoking**	**341 (94.7)**
**Depression/mental health**	**216 (60)**
**Being overweight**	**305 (84.7)**
**Alcohol**	**335 (93.1)**
**Steroids for muscle growth**	**323 (89.7)**
**Low sperm count**	**342 (95)**
Eating red meat	303 (84.2)
Lifting heavy weights	331 (91.9)
Using hot tubs	191 (53.1)
Multiple sexual partners	335 (93.1)
Frequent masturbation	318 (88.3)
Premature ejaculation	293 (81.4)
Height	359 (99.7)
Genital size	346 (96.1)

Bold shows factors that affect male fertility.

**Table 6:** Subgroup analysis—sexual orientation.

Does Male Depression Affect Fertility?			
Sexual Orientation	Correct	Incorrect	*p* Value
Asexual	0	2	0.018
Bisexual	22	5	
Heterosexual	183	132	
Homosexual	5	1	
Does having multiple sexual partners affect male fertility?			
Asexual	2	0	<0.001
Bisexual	24	3	
Heterosexual	298	17	
Homosexual	3	3	

## Data Availability

The datasets used during the current study are available from the corresponding author upon reasonable request.

## Supplementary Materials

The following supporting information can be downloaded at: https://www.mdpi.com/article/10.3390/ejihpe14080141/s1, File S1: Knowledge of fertility questionnaire.
