# Supplementary material for: What Do the General Public Know about Infertility and Its Treatment?
Source: Eur J Investig Health Psychol Educ. 2024 Jul 24;14(8):2116–25. doi: 10.3390/ejihpe14080141 (PMC11353598; doi:10.3390/ejihpe14080141)
Supplement: Supplementary file 1 [file ejihpe-14-00141-s001.zip › ejihpe-3109869-supplementary.pdf]

Questionnaire with answers:

1. **<u>Demographics:</u>**

How old are you? __________

Are you happy to tell us your gender? If so, what do you class as your gender?

Female
Male
Non-binary
Prefer not to answer
Other, please specify

Are you happy to tell us your sexual orientation? If so, what is your sexual orientation?

Heterosexual
Homosexual
Bisexual
Asexual
Prefer not to answer
Other, please specify

What is your ethnicity?

- Asian
- Black, Caribbean or African
- Mixed or multiple ethnic groups
- White
- Other ethnic group

Where do you currently reside?______________

What is your highest level of education?

No formal education
Primary education
Secondary education or high school
Vocational qualification
University education

2. **<u>Personal fertility history:</u>**

Do you have any biological children?

Yes
No

Are you currently trying to get pregnant?

Yes
No
Not applicable

Do you have a condition which affects your fertility?

Yes
No
Not sure

Do you have worries/concerns about conceiving in the future?

Yes
No

Not applicable

Do you know anybody that has had fertility treatment?
Yes
No

Have you had fertility treatment?
Yes
No

If yes to the above question then,
Have you had ovulation induction
Have you had IUI
Have you had IVF (In Vitro Fertilisation)/ICSI (Intracytoplasmic sperm infection)

Also if yes to the above question, then what year did you have fertility treatment? __________

Would you consider fertility preservation methods such as freezing your eggs or sperm?
Yes
No
Maybe
I have already done this
Not applicable

3. **Test your fertility knowledge**

Infertility is defined as the inability to achieve a pregnancy after a period of regular unprotected intercourse. How long do you think this period is?
6 months
12 months
15 months
18 months
24 months

How many days are in an average menstrual cycle?
21 days
23 days
25 days
28 days
32 days
35 days

Which days of the average menstrual cycle are considered the best days (most fertile) to have sexual intercourse in order to become pregnant?
Day 1-5
Day 6-9
Day 10-15
Day 16-22
Day 23-28

On what day, of an average 28-day menstrual cycle, does ovulation (an egg being released) occur?
Day 7
Day 10
Day 14
Day 20
Day 24

How often do you think you need to have sexual intercourse to maximise the chance of conception?
Everyday
Twice daily
Two to Three times a week
Once a week
Twice a month

How long can sperm survive in the female reproductive tract?
Around 1 day
Around 3 days
Around 5 days
Around 10 days
Around 15 days

Once an egg is released from the ovary (ovulated), how long is it capable of being fertilised by a sperm?
Around 6-12 hours
Around 12-24 hours
Around 24-48 hours
Around 48-72 hours
Around 72-96 hours

Do you think the age you try to get pregnant matters for women? Yes/no

If yes, then at what age do you think fertility really starts declining? 30

Do you think the age you try to get pregnant matters for men? Yes/no

If yes, then at what age do you think fertility starts declining? 40-45

4. **Causes of infertility**

Which of the following factors can negatively impact male fertility?

- Smoking
- Depression/mental health
- Alcohol
- Being overweight
- Using steroids for muscle growth
- Eating (a lot of red meat)
- Lifting heavy weights
- Using hot tubs
- Low sperm count
- Multiple sexual partners
- Frequent masturbation
- Premature ejaculation
- Genital size or shape
- Height

Which of the following factors can negatively impact female fertility?

- Smoking
- Depression/mental health
- Being overweight

- Having recurrent yeast infections
- Previous UTIs
- Eating (a lot) of red meat
- Polycystic ovarian syndrome
- Endometriosis
- Previous terminations of pregnancy
- Multiple sexual partners
- Hormonal contraception use
- Frequent masturbation
- Breast size
- Genital size or shape
- Height

Sexually transmitted infections (STIs) are a cause of infertility in both men and women. Many STI's cause structural damage to the tubes which carry the egg to the womb (the fallopian tubes) and the tubes which carry the sperm to the penis (the sperm ducts). What STI is the most likely to cause this damage?

Chlamydia
Gonorrhea
Syphilis
Bacterial Vaginosis
Herpes
Trichomoniasis
HIV

In a couple struggling to conceive, is the problem more likely due to male or female problems?
Male
Female
Equal

5. **Fertility treatment knowledge**

Approximately how many couples struggle with infertility worldwide currently?
Around 8 million
Around 28 million
Around 48 million
Around 88 million
Around 100 million

What is the average success rate of conceiving with one cycle of IVF?
24%
32%
48%
73%

How much on average does one cycle of IVF cost?
£200-£500
£500-£1000
£1,500-£5,000
£5,000-£7000

Since the first IVF birth in 1978, how many babies have been born using this process?
0.5 million

3 million
8 million
20 million

In the UK, what is the average number of NHS IVF funded cycles for couples struggling to conceive?
1
2
3
4
5
Do you think your health service should fund IVF?
Yes
No

If yes, how many cycles?
1
2
3
4
5
If not, who should fund? _______________

I feel like I received substantial teaching on fertility in school/college?
- Highly agree
- Agree
- Neutral
- Disagree
- Highly disagree

What would you like to have been taught?__________________
